# Evaluation of Death Anxiety in Patients with Schizophrenia

**DOI:** 10.1192/j.eurpsy.2026.10523

**Published:** 2026-07-31

**Authors:** H. Hsine, A. Bouaziz, M. Barkallah, S. Kolsi, W. Abbes

**Affiliations:** 1 Psychiatry, La Métairie Clinic, Nyon, Switzerland; 2 Psychiatry, Gabes university hospital, Gabes, Tunisia

## Abstract

**Introduction:**

Studies investigating death anxiety in patients with schizophrenia remain limited. This type of anxiety, fueled by distorted perceptions of reality and irrational fears, appears to be influenced by both individual and clinical factors.

**Objectives:**

our aim is to assess the level of death anxiety in patients with schizophrenia and to examine the factors associated with it.

**Methods:**

This was a cross-sectional, descriptive, and analytical study conducted among patients with schizophrenia attending the outpatient clinic of the Department of Psychiatry at the University Hospital of Gabès between January and February 2025. Sociodemographic and clinical data were collected using a pre-established information sheet. Schizophrenic symptoms were assessed using the PANSS (Positive and Negative Syndrome Scale), and death anxiety was measured using the TDAS (Templer Death Anxiety Scale).

**Results:**

A total of 90 patients were recruited. The mean age of patients was 46 years ± 12.9, with a male-to-female ratio of 2.3. Among them, 51.16% were single, 48.84% had a low socioeconomic status, and 37.21% were unemployed. The mean age of onset of illness was 23 ± 4.6 years. The mean number of hospitalizations per patient was 2 (ranging from 0 to 12), with an average hospitalization duration of 72.69 days. The mean total TDAS score was 6.28 ± 4.2, the mean total PANSS score was 76.47 ± 37.24, the mean positive PANSS score was 13.84 ± 6.76, the mean negative PANSS score was 18.44 ± 11.36, and the mean general psychopathology PANSS score was 44.19 ± 25.29.

The total TDAS score was significantly higher in patients with low socioeconomic status (p = 0.004) and those unemployed (p = 0.003). We found a positive correlation between the total TDAS score and positive PANSS scores (p = 0.001; r = 0.706), general psychopathology PANSS scores (p = 0.001; r = 0.577), and total PANSS scores (p = 0.001; r = 0.662).

**Conclusions:**

Our study shows that death anxiety among patients with schizophrenia seems to be linked to psychosocial vulnerability factors and influenced by psychotic symptoms. It is essential to better understand the nature of this anxiety, taking into account the associated factors, to ensure appropriate management.

**Disclosure of Interest:**

None Declared

